# Mind-Body Practice Changes Fractional Amplitude of Low Frequency Fluctuations in Intrinsic Control Networks

**DOI:** 10.3389/fpsyg.2017.01049

**Published:** 2017-07-07

**Authors:** Gao-Xia Wei, Zhu-Qing Gong, Zhi Yang, Xi-Nian Zuo

**Affiliations:** ^1^Key Laboratory of Behavioral Science, Magnetic Resonance Imaging Research Center, Institute of Psychology, Chinese Academy of SciencesBeijing, China; ^2^Department of Psychiatry, Massachusetts General Hospital, Harvard Medical School, BostonMA, United States; ^3^Lifespan Connectomics and Behavior Team, Institute of Psychology, Chinese Academy of SciencesBeijing, China; ^4^University of Chinese Academy of SciencesBeijing, China

**Keywords:** mind-body practice, frontoparietal network, fMRI, plasticity, amplitude, Tai Chi Chuan

## Abstract

Cognitive control impairment is a typical symptom largely reported in populations with neurological disorders. Previous studies have provided evidence about the changes in cognitive control induced by mind-body training. However, the neural correlates underlying the effect of extensive mind-body practice on cognitive control remain largely unknown. Using resting-state functional magnetic resonance imaging, we characterized dynamic fluctuations in large-scale intrinsic connectivity networks associated with mind-body practice, and examined their differences between healthy controls and Tai Chi Chuan (TCC) practitioners. Compared with a control group, the TCC group revealed significantly decreased fractional Amplitude of Low Frequency Fluctuations (fALFF) in the bilateral frontoparietal network, default mode network and dorsal prefrontal-angular gyri network. Furthermore, we detected a significant association between mind-body practice experience and fALFF in the default mode network, as well as an association between cognitive control performance and fALFF of the frontoparietal network. This provides the first evidence of large-scale functional connectivity in brain networks associated with mind-body practice, shedding light on the neural network changes that accompany intensive mind-body training. It also highlights the functionally plastic role of the frontoparietal network in the context of the “immune system” of mental health recently developed in relation to flexible hub theory.

## Introduction

Various neurological diseases associated with significant deficits in cognitive control have been widely reported in the literature (Verma and Howard, 2012; Robbins and Cools, 2014). Notably, cognitive control declines with the development of the disease and is not reversible. Therefore, the prevention of neurological diseases associated with declining cognitive control appears to be important and should become one of the best long-term strategies to solve this public health problem.

Currently, physiotherapeutic or exercise-related interventions are regarded as promising non-pharmaceutical tools to help improve cognition, especially in older at-risk individuals (Aas-Hansen et al., 2000). A recent meta-analysis concluded that exercise, particularly when meeting physical activity guidelines, can improve clinical symptoms in adults with neurologic disorders by enhancing cognitive control (Adamson et al., 2015). Based on the findings of several studies investigating the involvement of fronto-limbic regions after mindfulness meditation practice, it has been proposed that mindfulness works by strengthening prefrontal cognitive control mechanisms and thus down-regulates activity in regions relevant to affective processing (Tang et al., 2015). Therefore, integrative mind-body practice, consisting of both physical exercise and a meditation component, could have broader implications for the cognitive treatment of psychiatric and neurologic disorders. It is worth mentioning that Marciniak et al. (2014) proposed a theoretical framework on the effects of mindfulness-based practice in the context of aging and neurodegenerative diseases, which addressed the pivotal role of cognitive control among various effects, including physiological aspects (cholesterol) and the neuroprotection profiles of emotions.

Recent advances of resting-state MRI in functional and structural connectomics have revealed a complex interplay of different brain areas (i.e., instead of a set of brain areas with highly specialized functions) (Power et al., 2011; Bullmore and Sporns, 2012). These advances also highlight the role of macro-scale networks in the study of resting human brain function and its association with the mind, behavior, and disease. Several new findings related to training for physical exercise and mind have emphasized the importance of crucial functional networks such as default network, frontoparietal network, and salience network (Brewer et al., 2011; Krafft et al., 2014). Notably, recent findings suggest the existence of a frontoparietal control system, consisting of flexible hubs that regulate distributed systems throughout the brain, which is closely associated with mental diseases (Cole et al., 2014). It is also suggested that extensive training could reduce activity of the frontoparietal control system (Chiesa et al., 2013). Alternatively, with the help of similar mental or physical training, the frontoparietal control system could be responsible for training other systems to automatically facilitate an optimal state; a state incompatible with a variety of harmful dysfunctions. Accordingly, it is of great importance to investigate whether mind-body practice could optimize the activity pattern of the frontoparietal control system.

Currently, low frequency oscillations (typically defined as frequencies < 0.1 Hz) in resting-state have gained increased attention based on observations using fMRI approaches (Fox and Raichle, 2007). The fractional Amplitude of Low Frequency Fluctuations (fALFF) is defined as the total power within the frequency range between 0.01Hz and 0.1 Hz divided by the total power in the entire detectable frequency range, which is determined by sampling rate and duration (Zou et al., 2008). As a normalized index of ALFF, fALFF appears to be a biologically significant parameter for assessing regional brain function and can provide a more specific measure of low frequency oscillatory phenomena (Zuo et al., 2010), which has recently been widely employed in mental diseases studies.

Tai Chi Chuan (TCC) originated in China as an integrative form of aerobic exercise and meditation and is regarded as a typical mind-body practice (Mansky et al., 2006; Sharma and Haider, 2015). Used as a clinical treatment, TCC has demonstrated potential benefits for people with chronic neurological diseases such as PD (Allen et al., 2011), AD (Klein, 2008), multiple sclerosis (MS), and mood disorders (Payne and Crane-Godreau, 2013; Wang et al., 2014). On a behavioral level, several cross-sectional and longitudinal studies have confirmed the positive effect of mind-body practice, mainly on cognitive control. A preliminary study with a pre-to-post test design observed TCC practitioners had significantly improved performance on cognitive executive control after a 10-week TCC program (Matthews and Williams, 2008). In more recent work, we have demonstrated that a TCC group showed shorter trend than a control group in reaction time (RT) in flanker test and this was significantly associated with TCC experience among aging TCC practitioners (Wei et al., 2013). This evidence provides a theoretical rationale for exploring the relationship between optimized macro-level brain networks and cognitive control following extensive TCC practice.

Hence, examining the low frequency activity pattern in resting-state is a potential way to explore the optimized large-scale brain networks induced by TCC, especially the frontoparietal control system. This research strategy will allow us to deepen our understanding of TCC’s role in cognitive control and how that can be applied to clinical prevention and treatment of populations with neurological disorders. We hypothesize that frontoparietal control systems might be an important locus associated with TCC practice. Specifically, we predict that (1) we will find significant differences in fALFF in brain networks including frontoparietal network in experienced TCC practitioners compared with their matched controls; (2) these changes of fALFF in the frontoparietal network are behaviorally relevant to cognitive control in experienced TCC practitioners; (3) there is significant association between TCC experience and fALFF changes in practitioners’ brain networks.

## Materials and Methods

### Participants

We recruited TCC participants from a local community in Beijing. This group included 22 experienced TCC practitioners with a mean age of 52.4 ± 6.8 years old and a mean education level of 12.2 ± 2.9 years. On average, TCC participants had 14.6 ± 8.6 years of TCC experience and practiced 11.9 ± 5.1 h per week. One participant deviated from the group in terms of the intensity of TCC practice (30 h each week), and thus constituted an outlier. After removing this participant, the average years and intensity of TCC experience remained unchanged, but the estimation of practice hours (years × practice hours per day × days) changed from 9156 to 8775 h. This participant was kept in the following statistical analyses, including brain networks analyses and the correlation computation between cognitive performance and brain networks, to avoid losing any statistical power by decreasing the sample size. According to a demographic survey, participants practiced different styles of TCC including Yang, Sun, Wu, and Chen with varied overt movements. However, these styles still share essential components of TCC. The control group was matched with the TCC cohort by age, gender, and education (age: 54.8 ± 6.8 years old, years of education: 11.8 ± 2.9 years). None of controls had experience in any type of regular TCC, yoga, meditation or exercise practices.

The Institutional Review Board of the Institute of Psychology, Chinese Academy of Sciences approved this study. This study was performed in accordance with the Declaration of Helsinki and its later amendments. The procedure of the study was fully explained to the participants and informed written consent was obtained from each of them before the study. None of the participants had any history of neurological or psychiatric illnesses, injury, seizures, metal implants, head trauma with loss of consciousness and were not on any chronic medications that could affect the experiment.

### Cognitive Control Task

Ten participants separately from TCC group and control group completed the computerized Attention Network Test (ANT) (Fan et al., 2002) before fMRI scanning. In this study, we only report conflict effect (reaction time in inconsistent condition minus reaction time in consistent condition) during ANT that is target-related and reflects the performance of executive function. The experimental procedures have been previously described by Fan et al. (2002) and are only briefly introduced below. Each participant viewed a computer screen from a distance of around 50 cm after correcting for visual acuity. Eprime 2.0 professional (Psychology Software Tools, Pittsburgh, PA, United States) was used to program and present the task, which automatically collected all responses including reaction time and accuracy as soon as the participant completed the whole test. During the test, participants were instructed to respond as fast and accurately as possible via two input keys on a keyboard. The target stimulus was an arrow, indicating either the left or the right direction, presented in the center of a horizontal row of five arrows. The four surrounding flanker stimuli were all arrows pointing in the same direction as the central arrow (congruent condition) or the opposite direction of the target stimulus (incongruent condition) or were just neutral stripes (neutral condition). The target stimulus and the flanker stimuli were presented at a visual angle of 1.1 above or below a fixation cross presented in the middle of the screen. Preceding the presentation of the target, one of four cue conditions was provided: no cue; center cue; double cue; or spatial cue. These four conditions interact with the flanker task to influence RT. In brief, each trial consisted of the following structure: (1) a fixation cross was presented in the middle of the screen during a variable interval, ranging from 400 to 1600 ms; (2) a 100 ms cue was presented; (3) a 400 ms central fixation was presented; (4) the target stimulus was presented for 1700 ms, or shorter if a response was given within 1700 ms; (5) a fixation cross was presented during a variable delay, which of this delay was determined by subtracting the RT plus 400 ms from the total trial duration being kept constant at 3500 ms.

The experiment lasted approximately 25 min and involved 288 trials, which were divided into three blocks of 96 trials each with a short break between blocks. Before the main task, all participants completed a training block of 24 randomly selected trials, which provided feedback for accuracy and RT at the end of each trial. This ensured that participants completely understood the task instructions.

### Scanning Protocols

Brain imaging was performed on a 3T Trio system (Siemens, Erlangen, Germany) with a 12-channel head matrix coil. Two hundreds and forty-three volumes were obtained by using an echo planar imaging (EPI) sequence with the following scan parameters: repetition time (TR) = 2000 ms, echo time (TE) = 30 ms, flip angle (FA) = 90^o^, slice thickness = 3.0 mm, field of view (FOV) = 200 mm^2^ × 200 mm^2^, voxel-size = 3.4 mm × 3.4 mm × 4.0 mm, resulting in 243 brain volumes of 30 axial slices. During the resting scans, all participants were instructed to keep their eyes closed, relax, and move as little as possible. We also acquired a three dimensional magnetization prepared rapid gradient echo (3D MPRAGE) sequence for anatomical information with the resolution of of 1.3 mm × 1.0 mm × 1.3 mm (TR = 2530 ms, TE = 3.39 ms, FA = 7^o^, slice thickness = 1.33 mm) for better registration and overlay of brain activity.

### Image Preprocessing and Quality Control

#### Preprocessing Steps and QC Procedures

Preprocessing of the structural and functional images was implemented with the Connectome Computation System^1^ (CCS) (Zuo et al., 2013; Xu et al., 2015), which is a computational platform for brain connectome analysis with integrating FreeSurfer (Dale et al., 1999; Fischl et al., 1999), FSL and AFNI to provide a pipeline system for multimodal image analysis. The data preprocessing was composed of steps for both anatomical and functional processing.

The structural image processing included the following steps of brain cortical surface reconstruction (Dale et al., 1999; Fischl et al., 1999; Segonne et al., 2004, 2007). Firstly, MR image noise was removed by using a spatially adaptive non-local means filter and MR intensity heterogeneity correction (Xing et al., 2011; Zuo and Xing, 2011). Brain was extracted with a hybrid watershed/surface deformation procedure and was segmented into different tissues such as the cerebrospinal fluid (CSF), white matter (WM), and deep gray matter (GM) volumetric structures. A triangular mesh tessellation was estimated over the GM-WM boundary and the mesh was deformed to produce a smooth representation of the GM-WM interface (white surface) and the GM-CSF interface (pial surface) spatial normalization from individual native space to *fsaverage* stereotaxic space. Correcting topological defect on the surface and inflated individual surface mesh into a sphere. Finally, making estimation of the deformation between the resulting spherical mesh and a common spherical coordinate system.

The functional image preprocessing discarded the first five EPI volumes from each scan to allow for signal equilibration, removed and interpolated temporal spikes, corrected acquisition timing among image slices and head motion among image volumes, normalized the 4D global mean intensity to 10,000 to allow inter-subject comparisons and regressed out the WM/CSF mean time series and the Friston-24 motion time series to reduce the effects of these confounding factors (Yan et al., 2013; Zuo et al., 2013). Finally, the residual time series with a band-pass (0.01–0.1 Hz) were filtered to extract the low frequency fluctuations. Both linear trends and quadratic trends were removed and individual motion corrected functional images were aligned to the individual anatomical image using the GM-WM boundary-based registration (BBR) algorithm (Greve and Fischl, 2009).

By combining BBR deformation and spherical surface normalization, the individually preprocessed 4D rfMRI time series were projected onto the *fsaverage* standard cortical surface with 163,842 vertices per hemisphere, with an average distance of 1 mm for neighboring pairs of vertices. Then, the data were down-sampled onto the *fsaverage5* standard cortical surface (3.8-mm neighboring-vertex distance), which contained 10,242 vertices per hemisphere (Yeo et al., 2011).

#### Quality Control Procedure

After preprocessing individual images, quality control procedure (QCP) was conducted by using CCS. Specifically, this procedure produces basic information concerning preprocessed images, including screenshots for visual inspection of: (1) skull stripping, (2) segmentation of brain tissue, (3) reconstruction of pial and white surface, (4) registration of BBR-based functional image, and (5) head motion processing during rfMRI. Several quantities are also produced, including the following: (1) the maximum distance of translational head movement (maxTran), (2) the maximum degree of rotational head movement (maxRot), (3) the mean frame-wise displacement (mean FD) (Power et al., 2012; Patriat et al., 2013), and (4) the minimal cost of the BBR co-registration (mcBBR). Any participants with bad brain extraction, tissue segmentation, and bad surface construction will be excluded from the subsequent analysis. Moreover, all datasets in the subsequent analysis must meet some criteria, which is described in detail of the website^2^.

### Computation of Network-Level fALFF

**FIGURE 1:**
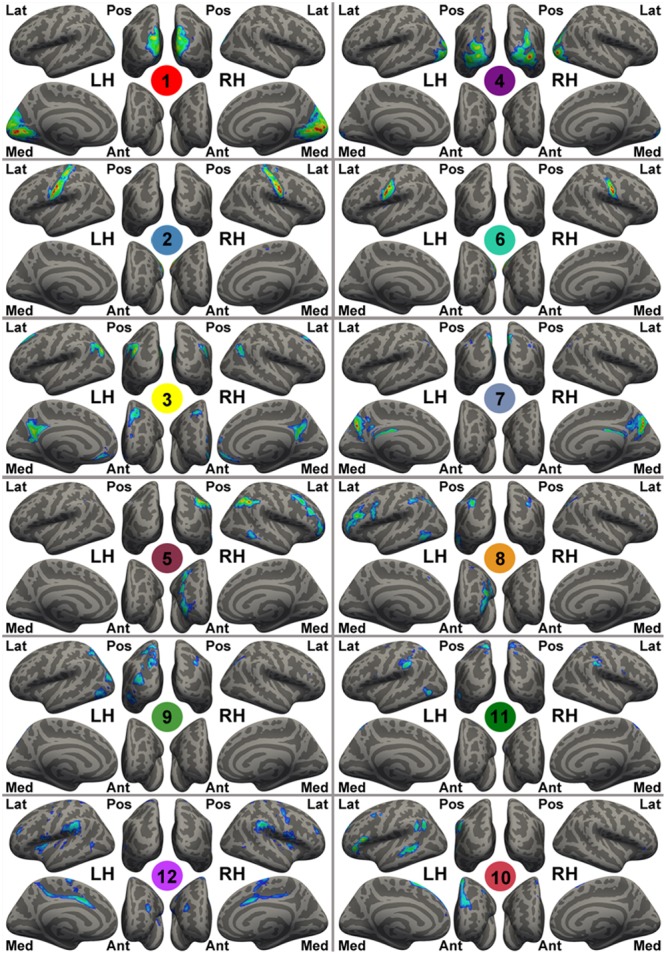
Template intrinsic connectivity network (ICN) maps generated by gRAICAR using the NKI-RS sample. For the purpose of visualization, these template maps are rendered onto fsaverage standard surfaces from lateral (Lat), medial (Med), posterior (Pos), anterior (Ant) views in Freesurfer for both left (LH) and right (RH) hemispheres and thresholded at | Z| > 1. The *Z*-value here is the original intensity of the maps from gRAICAR (i.e., weighted *z*-scores given by MELODIC), instead of the value after spatial standardization. These ICN templates are labeled with different colorful circles and numbers at the center of the circles, representing: the primary visual cortex (medial occipital lobe, ICN01), bilateral primary motor network (ICN02), the default mode network (ICN03), the lateral posterior occipital cortex (ICN04), the right-lateralized frontal-parietal network (ICN05), bilateral ends of the central sulcus (ICN06), the precuneus-dorsal posterior cingulate network (ICN07), the left-lateralized frontal-parietal network (ICN08), the dorsal precuneus-bilateral angular gyri network (ICN09), the anterior cingulate-dorsal prefrontal-angular gyri network (ICN10), the dorsal precuneus-bilateral temporal network (ICN11), and the bilateral superior temporal-inferior frontal network (ICN12).

A set of spatial templates of 12 common intrinsic connectivity networks (ICNs) was generated from an independent sample, the NKI-rockland sample (*N* = 126), using an exploratory group-level intrinsic network discovery tool, gRAICAR (Yang et al., 2014; **Figure 1**). Data from the NKI-rockland sample were preprocessed using the same CCS pipeline. The preprocessed functional images were processed using gRAICAR (Yang et al., 2008, 2012) to characterize the consistency of the ICNs across all of the subjects. Spatial independent components (ICs) were derived from each subject using the MELODIC module of FSL, where the number of independent components was automatically determined. All of the ICs from all the subjects were pooled in gRAICAR, and normalized mutual information between every pair of ICs was computed to yield a full similarity matrix. The full similarity matrix was then searched to match ICs across different subjects, forming group-level aligned components (ACs). Each AC was formed by a set of matched ICs containing one IC from each subject. For each of the ACs, a similarity matrix was computed to reflect the similarity between its comprising ICs, each representing a subject. In the inter-subject similarity matrix, the centrality of a subject’s IC was computed by summing up the similarity metrics between that subject’s IC and all other ICs in that AC. The significance of the centrality of each subject was examined using a permutation test. The centrality measures were then used as weights to average the spatial maps of the constituent ICs into a group-level spatial map for that AC. In summary, the spatial maps of the ACs represent ICNs or artifacts in the resting-state data, and the similarity matrices reveal inter-subject consistency of the ACs. According to the permutation test of inter-subject consistency, 12 ICN maps were (significantly) consistent across >60% of the subjects, and therefore these ICNs were used as ICN templates for further study.

Representative time series of these ICNs were obtained in both the original data and the band-pass (0.01–0.1Hz) filtered data by using spatial regression. The power of the representative time series from band-pass filtered data was divided by that of the original data, yielding fALFF of each ICN, a measure of the network dynamics or variability.

The fALFF metrics, demographic, and behavioral statistics were compared between TCC experts and control subjects using two sample *t*-tests in SPSS 20. Using SPSS, we conducted partial correlation analyses controlling for age, sex, and education between demographic data, behavioral data, and brain networks with significant differences. Only 21 TCC participants were involved in the correlation between TCC practice and brain networks due to the exclusion of an outlier based on practice hours each week.

## Results

### Participant Demographics

Participant demographic data are provided in **Table 1**. The results showed that there was no significant difference in all relevant variables (i.e., gender, age, and education) between the TCC group and the control group. We also computed intracranial volume, global fALFF, and the root mean square of frame-wise displacement parameter (indicating head motion), and found no significant difference between the two groups.

**Table 1:** Participant characteristic.

	*TCC Experts (N = 22)*	*Healthy Controls (N = 18)*	*p*
Age (Years)	52.4 ± 6.8	54.8 ± 6.8	0.258
Gender (Males/Females)	7/15	8/10	0.425
Education (Years)	12.2 ± 2.9	11.8 ± 2.9	0.666
TCC Duration (Years)	14.6 ± 8.6	NA	NA
TCC Intensity (Hours/Week)	11.9 ± 5.1	NA	NA
ICV^1^ (Liter)	1.11 ± 0.17	1.12 ± 0.22	0.42
Global fALFF	0.45 ± 0.03	0.44 ± 0.03	0.38
rmsFD^3^ (mm)	0.16 ± 0.09	0.12 ± 0.07	0.16

*^1^ICV, the intracranial volume; ^2^Global ReHo, Global mean regional functional homogeneity; ^3^rmsFD, root mean square of frame-wise displacements.*

### Cognitive Control Performance

**FIGURE 2:**
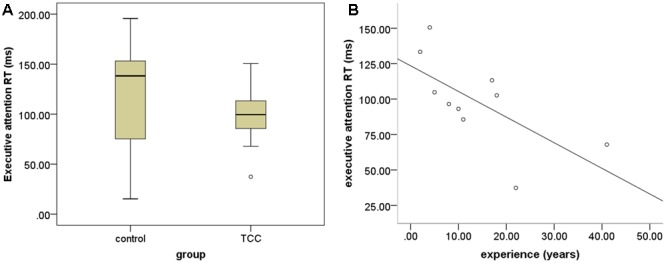
Group differences in behavioral task performance. **(A)** Group differnces in performance of executive attention between Tai Chi Chuan (TCC) group and control group; **(B)** scatter plot of the association between performance of executive attention and TCC experience (years of practice).

Response speed and accuracy are two important factors in assessing ANT performance. We computed the RT and accuracy in the cognitive control to examine the differences of executive function for the TCC group and the control group (see **Figure 2**). The two-sample *t*-tests revealed that the TCC group trended toward shorter RTs of cognitive control in ANT performance than the control group, although this difference was not significant. No significant group difference in accuracy of cognitive performance was detected. To examine the association between TCC practice and cognitive control, we also computed the correlation between these two factors. This showed that RT of cognitive control was negatively correlated with TCC experience (*r* = -0.659; *p* = 0.038).

### Group Differences in fALFF among Brain Networks

To test the hypothesis that TCC practitioners might show differential changes in cognitive control-related brain networks relative to controls, we performed a MANOVA analysis controlling for gender, age, and education (see **Table 2**). It was observed that the default network (ICN03) significantly decreased in fALFF for the TCC group compared with the control group (*F* = 5.344, *p* = 0.027). The bilateral frontoparietal network [right FPN (ICN05): *F* = 5.491, *p* = 0.025; left FPN (ICN08): *F* = 12.963, *p* = 0.001] revealed significantly decreased fALFF in the TCC group compared with controls. The anterior cingulate-dorsal prefrontal-angular gyri network (ICN10) in the TCC group also showed a trend of significantly decreased fALFF relative to the control group (*F* = 4.108; *p* = 0.05).

**Table 2:** Group difference of fractional Amplitude of Low Frequency Fluctuation (fALFF) after controlling gender, age, and education as covariates.

	CTR (*n* = 18)		TCC (*n* = 22)		*t*	*p*
	*M*	*SD*	*M*	*SD*		
ICN01	0.738	0.125	0.724	0.145	0.117	0.907
ICN02	0.655	0.117	0.611	0.173	0.793	0.433
ICN03	0.831	0.075	0.751	0.124	2.312	0.027^∗^
ICN04	0.692	0.117	0.699	0.151	-0.396	0.695
ICN05	0.685	0.087	0.614	0.123	2.343	0.025^∗^
ICN06	0.602	0.111	0.545	0.168	1.084	0.286
ICN07	0.738	0.084	0.700	0.095	1.170	0.250
ICN08	0.765	0.079	0.672	0.095	3.600	0.001^∗∗^
ICN09	0.690	0.103	0.679	0.138	-0.049	0.961
ICN10	0.677	0.084	0.599	0.146	2.027	0.050
ICN11	0.662	0.114	0.660	0.136	0.232	0.818
ICN12	0.718	0.075	0.670	0.122	1.285	0.207

*^∗^Indicated *p* < 0.05.*

*^∗∗^Indicated *p* < 0.01.*

### Association between Behavioral Performance and Brain Networks

**FIGURE 3:**
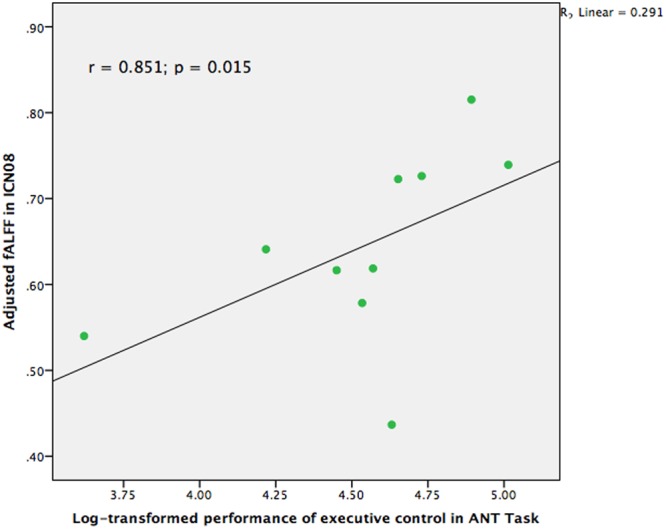
Scatter plot indicating the correlation between fractional Amplitude of Low Frequency Fluctuation (fALFF) in ICN08 and log-transformed performance of executive control in Attention Network Test (ANT) task controlling for age, gender, and education (*r* = 0.851; *p* = 0.015).

Correlational analyses were also conducted to examine whether the group differences among those brain networks were also related to the performance of cognitive control in the ANT task in the group of TCC practitioners. Partial *r* correlation coefficients were computed between the RT and the three fALFF values of the brain networks which were significantly different in the between-group comparisons, to avoid the occurrence of false positive results. We also performed partial correlation analyses between accuracy of the ANT task and the fALFF values in the three brain networks. Logarithmic transformation was also conducted for RT and accuracy of the ANT task since the values of these two groups were distributed non-normally. As **Figure 3** indicates, the results demonstrated that ICN08 was significantly correlated with RT of the ANT task (*r* = 0.851; *p* = 0.015) while no significant correlation was observed in the association between accuracy of the ANT task and brain networks.

### Association between TCC Practice and Brain Networks

**FIGURE 4:**
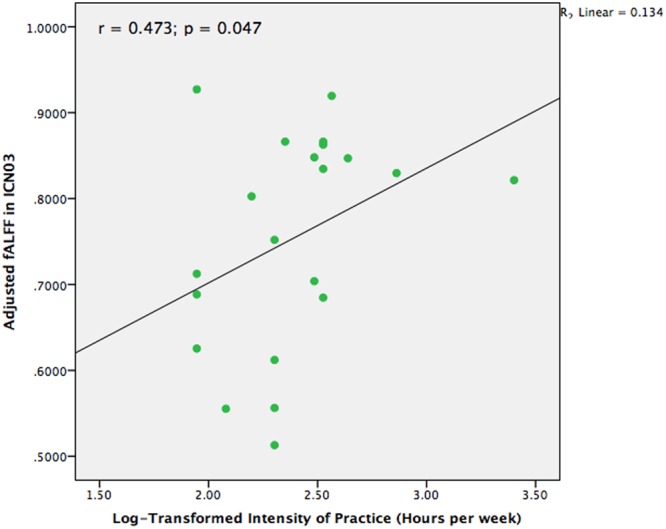
Scatter plot to indicate the correlation between fALFF in ICN03 and intensity of TCC practice after controlling for age, gender, and education (*r* = 0.473; *p* = 0.047).

In our previous structural study on TCC practitioners, we found that intensity of practice is a valid and sensitive indicator of TCC experience (Wei et al., 2013). Hence, intensity of practice was also adopted in the present study to test the different fALFF among the brain networks in the TCC group that might be associated with TCC experience. We performed a partial correlation analysis between TCC experience and fALFF of brain networks controlling for age, sex, and education. The values for intensity of TCC practice were log-transformed for marginally non-normally distributed trend (Shapiro–Wilk Test, *p* = 0.056). It is likely that non-linear relationship may exist in the effect of TCC practice on brain networks. The normality of intensity scores of TCC practice was improved with this transformation (Shapiro–Wilk test, *p* = 0.119). Moreover, one participant had practiced TCC for at least 30 h each week, which is an outlier based on a distributed scatter plot of the descriptive data. We removed this participant from the analysis for calculating the correlation between the remaining 21 practitioners’ fALFF in brain networks and intensity of TCC practice. As **Figure 4** indicates, we observed that the fALFF of ICN03 significantly correlated with the log-transformed intensity of practice (*r* = 0.473, *p* = 0.047). No other significant correlations based upon the transformed intensity in the significantly different brain networks between groups were detected.

## Discussion

To our knowledge, this is the first study to specifically examine the association between mind-body practice and large-scale brain networks. In this study, to investigate practice-induced resting low frequency activity in large-scale brain networks, we used a recently developed measure of inter-subject reproducibility-based algorithms for detection of functional brain networks – gRAICAR. Firstly, we compared the fALFF of brain networks between groups, which showed significant decreases of fALFF in the bilateral frontoparietal network (ICN08 and ICN05) in the TCC group compared to the control group. Additionally, fALFF values in the default mode network (ICN03) and the dorsal prefrontal-angular gyri network (ICN10) were also greatly decreased in practitioners relative to controls. Secondly, we aimed to explore whether extensive TCC practice induced the changed fALFF in brain networks. The results revealed associations between the left lateral FPN and performance of cognitive control, as well as an association between the default mode network and practice experience. This indicates the positive impact of extensive TCC practice on cognitive control-related brain systems.

### Functional Plasticity Associated with Mind-Body Practice

This investigation exploring the altered intrinsic cortical network associated with mind-body practice showed that the effects of extensive mind-body training on brain networks were rather selective, being largely located in the frontoparietal, default mode, and dorsal prefrontal-angular networks. The alteration on the amplitude of low frequency fluctuation in several brain networks, including frontoparietal network, strongly supports our hypothesis. It is suggested that the difference of fALFF in brain networks between the TCC group and the control group possibly reflect experience-dependent neural plasticity. Neural plasticity is the ability to make adaptive changes related to structure and function of the nervous system (Zilles, 1992). Numerous studies on animal models and human species have suggested that training is a key environmental factor to induce morphological alterations in brain areas, changes in neuron morphology, network alterations (including changes in neuronal connectivity), the generation of new neurons, and neurochemical changes (Fuchs and Flugge, 2014). Consistent with this result, previous studies have reported the selective effects of training on brain networks or regions.

#### Mind-Body Practice and Frontoparietal Network

Evidence from young adults has revealed that intensive reasoning training was associated with increased frontoparietal connectivity (Mackey et al., 2013). Moreover, 2 weeks working memory training was reported to alter the activity pattern of frontoparietal network (Schneiders et al., 2012). Although there are relatively few studies directly focused on the change of FPN associated with mind-body practice, several studies consistently observed such an effect on cognitive control-related brain activity involving prefrontal and parietal cortex. A cross-sectional study on yoga practitioners observed less reactivity in right dorsolateral prefrontal cortex (involving attention and exerting cognitive control-related function) to negative images compared to neutral images (Froeliger et al., 2012), which indicated the top-down modulation of PFC on emotional regulation by mind-body practice. Recent neuroimaging evidence also found that mindfulness practitioners revealed decreased frontal activation during processing of emotionally aversive experiences (Gard et al., 2012). These findings likely reflected the attitude of acceptance and non-judgment without effortful cognitive control during emotional processing developed after extensive practice. Additionally, mind-body intervention studies using EEG indicated the critical role of prefrontal cortex in cognitive improvement among people with autism and choric epilepsy (Chan et al., 2009, 2011). Similarly, another randomized controlled study showed that Chinese chan-based mind-body intervention significantly improved frontal alpha asymmetry and intra- and inter-hemispheric theta coherence in frontoposterior and posterior brain regions among patients with major depressive disorder (Chan et al., 2013).

Notably, it is well established that the change in functional activity at the cortical level following intensive physical or mental training largely involves prefrontal and parietal regions (based on brain “location specific” approach). The functional plasticity of prefrontal and parietal cortex has been well documented in mindfulness and physical exercise studies, respectively. For instance, following 6 weeks of mindfulness training, dorsolateral PFC responses were increased during executive processing in an emotional Stroop task in healthy human participants (Allen et al., 2012), which indicated such training benefits the recruitment of a top-down mechanism to resolve cognitive conflict. A study showed that greater activation in parietal regions were also found after meditation training in groups with social anxiety (Goldin and Gross, 2010). By contrast, meditation experts were characterized by decreased activation in dorso-and ventrolateral PFC regions compared with controls in cross-sectional studies (Grant et al., 2011; Gard et al., 2012). These findings could be explained by the different demands of cognitive control between the beginning and expertised practice stages. Moreover, short-term mindfulness training studies also observed increased EEG power in the theta frequency at frontal midline electrodes (Tang et al., 2009). Regarding the alteration of brain structures induced by meditation a recent meta-analysis of morphometric neuroimaging in meditation examining approximately 300 meditation practitioners demonstrated that prefrontal cortex showed the most consistent differences between meditators and controls (Fox et al., 2014).

Additionally, convincing evidence from physical exercise studies has demonstrated the effect of physical activity on cognitive control, as well as on prefrontal and parietal cortex (Loprinzi et al., 2013). EEG studies have shown increased neural activity within the prefrontal cortex and improved executive functioning performance following acute physical activity (Hillman et al., 2003). This finding was also confirmed with other cognitive control paradigms including a switching task and an attentional network test, which showed either reduced latency of neural activity or larger P3 amplitude in the prefrontal and parietal cortices. These results suggest the role of physical activity in improving cognitive performance through mechanisms related to cognitive control (Kamijo and Takeda, 2010; Chang et al., 2015). A recent review has also pointed out the involvement of FPN in initiation and flexible adjustments in cognitive control during engaging physical activity behavior (Buckley et al., 2014).

#### Mind-Body Practice Experience and Default Mode Network

To understand the question of whether mind-body practice resulted in the change of DMN, we compared the difference of fALFF in DMN between two groups and assessed the relationship between DMN changes and practice experience. The results showed that the fALFF in DMN was significantly different in TCC practitioners relative to controls and were positively correlated with practice intensity (practice hours/week). The association between experience and changed fALFF in DMN generally reflected experience-dependent functional plasticity, which confirmed the effect of mind-body practice on the functional pattern of DMN. Currently, an increasing amount of evidence consistently demonstrates a pattern of deactivation during a task-invoked state as well as activation across a network of brain regions during resting-state, including medial, lateral, and inferior parietal cortex, precuneus/posterior cingulate cortex (PCC) and medial prefrontal cortex, and (Raichle et al., 2001). This network is defined as the default mode network and is characterized by coherent low frequency neuronal oscillations, which reflect the associated psychological functions of introspection and self-referential thought (Broyd et al., 2009). An extensive body of research defines the DMN to be one of the critical networks of the human brain. It can also be altered by various practices. Consistent with this study, prior investigations of the link between large-scale brain network pattern and physical or mental training have also pointed to the importance of the default mode network. Voss et al. (2010a) adopted a seed-based functional connectivity analysis examining the association between aerobic fitness, cognitive performance and functional connectivity in the default mode network, which concluded that both specific and global default mode networks mediated the relationship between aerobic fitness and cognition. Furthermore, another 12-month randomized interventional study also observed that exercise training increased functional connectivity between some brain regions within the default mode network in elderly adults. This provided the first evidence for exercise-induced functional plasticity in large-scale brain systems in the aging brain (Voss et al., 2010b). In parallel to this, the evidence from mindfulness studies has consistently detected training-induced functional and structural changes in DMN, which suggests DMN plays a pivotal role in processes of internal mentation. For example, a cross-sectional study among meditators with extensive training demonstrated that meditation training can lead to functional connectivity changes between core DMN regions, possibly reflecting strengthened present-moment awareness (Taylor et al., 2013). Meditation training has also been observed to increase the functional connectivity within DMN in elderly adults with mild cognitive impairments (Wells et al., 2013), which could be interpreted as a role of DMN of attenuating cognitive aging. A recent review on meditation suggested DMN is a biomarker for monitoring the therapeutic effects of meditation in mental disorders (Simon and Engstrom, 2015).

Tai Chi Chuan is a typical mind-body practice that is performed using a series of graceful concentric and eccentric movements that are linked together in a continuous sequence in semisquat positions. During the performance of TCC, deep breathing, slow movements, and mental concentration are required to achieve harmony between body and mind. Thus, TCC practice combines key components of various practices such as aerobic exercise and meditation (Yeh et al., 2014). Integrating these training factors may produce multiple effects. Thus, the findings of FPN and DMN changes in our study contribute to the multiple outcomes of long-term TCC practice, combining components of aerobic exercise and mindfulness.

### FPN, Possible Neural Correlate Underlying the Effect of Mind-Body Practice on Cognitive Control

In this study, we observed that the TCC group had decreased fALFF in bilateral FPN as well as an association between cognitive control performance and fALFF in FPN. Alternatively, TCC practice optimizes the spontaneous activity of FPN, coupling with enhanced cognitive behavior performance. It is plausible that an optimized pattern of FPN might play a role in the effect of mind-body practice on cognitive control. Although further mediation analysis didn’t show any significance to confirm fALFF in FPN mediated the effect of TCC practice on cognitive control, it is reasonable to infer that low frequency oscillation of regional brain function in FPN might be neural correlate underlying the effect of TCC practice on behavioral performance since FPN has highly flexible and variable connectivity throughout the brain, the functional connectivity of FPN with other brain systems and the global brain system. Given that a system refers to a set of widely distributed brain regions that exhibit consistently correlated spontaneous activity fluctuations, and characteristically respond toward a specific task, the brain is organized into multiple systems that have distinct and potentially competing functional roles. Recently, several big sample studies, across multiple datasets that aimed to explore the organization of human cerebral cortex estimated by intrinsic functional connectivity, have identified the frontoparietal control system as supporting cognitive control, including middle and superior prefrontal cortex (BA6, BA8, BA46, BA47, and BA10), superior parietal lobule (BA47), and inferior parietal lobule (BA 40) (Vincent et al., 2008; Yeo et al., 2011). Regions within the frontoparietal network showed similar functional connectivity fingerprints even when distributed across the cortex. Previous task-fMRI studies also indicated that many regions in the frontoparietal control system are activated during tasks requiring cognitive control or executive function (Hon, 2007). Furthermore, it is pointed out that this control system is also involved in integrating information coming from the other systems and to adjudicate between potentially competing inner- versus outer-directed processes (Vincent et al., 2008). Convincing evidence suggests that the human ability to adaptively implement a wide variety of tasks is primarily a result of the operation of the frontoparietal brain network. More recently, Cole et al. (2013) found that FPN’s brain-wide functional connectivity pattern shifted more than those of other networks across a variety of task states and that these connectivity patterns could be used to identify the current task. It was further confirmed that the frontoparietal network implements domain-general functions (e.g., the cognitive control system) made by flexible hubs (Cole et al., 2013). In view of the flexible hubs largely existent in FPN, we infer that extensive mind-body training exerts a preferential influence on general cognitive control among other multiple specific effects including self-awareness, self-regulation, proprioception, and goal planning. Notably, it has been emphasized that cognitive control showed the largest benefit of improved fitness among the different process-task types induced by physical exercise (Colcombe and Kramer, 2003; Chang et al., 2010). On a behavioral level, previous studies have observed such an effect of cognitive control following short-period TCC practice (Matthews and Williams, 2008). Intriguingly, our study also revealed that the TCC group showed marginally significant better performance than the control group. Moreover, the performance of cognitive control in the TCC group was correlated with TCC experience. These results further demonstrate the critical role of cognitive control during TCC practice. Hence, FPN, being responsible for general cognitive control, showed greater difference of regional brain function induced by mind-body practice relative to other brain systems.

### Enhanced Cognitive Control Capacity via Multiple Feedbacks As Outcomes of TCC Practice

As stated above, based on the evidence from neuroimaging approaches, a wide variety of mental disorders involve impaired cognitive control abilities and altered function in control system. Cole et al. (2014) suggested the mechanism of mental health using the framework of brain feedback control: a control system consisting of flexible hubs that use feedback control to regulate symptoms and so promote mental health. Hence, an effective control system would be protective against a variety of mental diseases. In view of the findings of our study, we propose that TCC practice is an efficient means to reach the goal of successful feedback control via the flexible hubs of the frontoparietal network. Then how does this mind-body practice execute this function among multiple and complex brain systems? More generally, cognitive control capacity can vary substantially both within and across individuals. The reduction of the control system might be influenced by excessive stress, cognitive load, and negative affect. By contrast, motivation, effective strategies for the goal, and adaptive habits could increase cognitive control capacity (Cole et al., 2014). It is likely that TCC could enhance the cognitive control system to perform indirect feedback control via multiple strategies such as deep breathing, mindfulness, and attention control, which is supported by relevant TCC studies on mental diseases. Previous short-period intervention studies suggested that TCC could be regarded as a treatment strategy to promote cognitive function in adults with cognitive impairment (Li et al., 2014) and cerebral vascular disorder (Wang et al., 2010), as well as depression, anxiety, and sleep disturbance (Field et al., 2013). We speculate that some health-related key components contained in TCC practice represent multiple channels of feedback, which accumulatively enhance cognitive control effects as outcomes of TCC practice.

Firstly, deep breathing, one of the key components of TCC, is increasingly used for its relaxation effect as a complementary and alternative medicine for maintaining general health, as well as treating myriad diseases. Several studies investigating TCC have confirmed that TCC practice could increase vagal activity and the balance between sympathetic and parasympathetic activity via increasing heart rate variability (HRV) (Lu and Kuo, 2003, 2014). We have also previously observed improved vagal modulation during deep breathing by comparing the HRV of TCC practitioners and controls (Wei et al., 2016). Although the underlying neural mechanism is unclear as to how deep breathing establishes the temporary neural circuits relevant to the control system, we speculate that TCC practice is beneficial to modulating the cognitive control system by increasing functional connectivity between the frontoparietal network and the corresponding cortical and subcortical structures dominating deep breathing. Meditation studies have reported that some brain regions which have stable anatomical and functional connectivity with the frontoparietal network, such as anterior cingulate cortex and insula, are responsible for the activity of the autonomic nervous system (Tang et al., 2009). Hence, it is likely that the functional connectivity between the frontoparietal network and these brain regions contributes to the execution of indirect feedback toward the control system. Secondly, attention control is also considered one of the key characteristics of TCC for the requirement of harmony between movement and mental activity. There is growing evidence that mindful meditation could induce functional changes of dorsal lateral prefrontal cortex and anterior cingulate cortex supporting attention control processes (Tang et al., 2015). Prefrontal cortex is the critical flexible hub existing in the frontoparietal network (Cole et al., 2013), while anterior cingulate cortex has relatively close connections in anatomical and functional connectivity with frontoparietal network. This partly determines the role of this component of TCC in the feedback channel. Thirdly, aerobic exercise, the last but not least component of TCC, has been extensively demonstrated to change GM volume (Colcombe et al., 2006), cortical thickness (Wei et al., 2013), and regional brain function (Colcombe et al., 2004) at the cortical level in prefrontal and parietal cortex, which is consistently implicated in cognitive control processing. In view of a long-term strategy, TCC practice might facilitate such established, long-term, body feedback by these specific brain regions to execute control-related feedback. Taken together, multiple feedback channels benefited by extensive TCC practice might be one of the key correlates underlying enhance cognitive control capacity to further perfect the “immune system” of mental health.

### Limitations

The results and interpretations of this study must be considered with several limitations. One limitation is that the cross-sectional examination between mind-body practice and brain networks could not completely exclude the effect of some confounding factors such as predisposition, preexisting characteristics of brain structure and function, and intelligence, although we controlled for age, gender, and education level when examining the group differences in fALFF and the correlation between behavior and the altered brain networks. Longitudinal studies will help delineate the beneficial effect of mind-body practice on resting brain state and will be needed in future research. Second, the findings of this study should be interpreted with caution given the relatively small sample size. In particular, the data of half the sample’s behavioral performance had not been collected, which possibly covers up more significant correlations in other brain networks. Third, although several clinical studies using gRAICAR showed good reliability in subject grouping, it still needs examination in normal populations with little variability compared to patients diagnosed for neurological diseases. Thus, an important area of future study could utilize this brain parcellation on the ICN to carry on the investigation in a normal population. Hopefully, it is promising to apply this to a longitudinal prevention study on mind-body practice.

## Conclusion

In sum, the present investigation firstly jointly employ MRI and behavioral methodologies to examine the link between mind-body practice and large-scale brain networks. A clear association between the cognitive control and frontoparietal networks is demonstrated. It is inferred that frontoparietal networks is likely to be the neural correlates underlying the effect of TCC practice on cognitive control. The results also extend previous research that has been based on a brain-specific location approach. The decreased fALFF in the left-lateralized frontoparietal network in the TCC practice group relative to the control group might reflect the improved cognitive control capacity associated with TCC practice. Other functional networks including the default network, the right-lateralized frontal-parietal network, and the dorsal prefrontal-angular gyri network are also an indication of functional optimization possibly induced by mind-body practice. Furthermore, the findings also carry significant public health implications. Although this study did not focus on patients with cognitive impairment, the optimized functional pattern in the frontoparietal network still help to unravel the partial neural correlates underlying the effect of mind-body practice on enhancing cognitive control capacity. This will hopefully encourage government to consider mind-body intervention in the treatment and prevention of illness and disease.

## Author Contributions

G-XW designed the work, drafted and finalized manuscript; Z-QG, ZY, and X-NZ analyzed data and revised manuscript.

## Conflict of Interest Statement

The authors declare that the research was conducted in the absence of any commercial or financial relationships that could be construed as a potential conflict of interest.

## Footnotes

1 https://github.com/zuoxinian/CCS

2 http://lfcd.psych.ac.cn/ccs/QC.html
